# Epidemiological characteristics and factors affecting length of hospital stay for children and adults with burns in Zunyi, China: a retrospective study

**DOI:** 10.7717/peerj.5740

**Published:** 2018-10-03

**Authors:** Tao Wang, Chan Nie, Hong Zhang, Xueqin Zeng, Huiting Yu, Zairong Wei, Chenglan Yang, Xiuquan Shi

**Affiliations:** 1 Department of Epidemiology and Health Statistics, Zunyi Medical University, Zunyi, Guizhou, People’s Republic of China; 2 Center for Medical Records, Affiliated Hospital of Zunyi Medical University, Zunyi, Guizhou, People’s Republic of China; 3 Burns & Plastic Surgery, Affiliated Hospital of Zunyi Medical University, Zunyi, Guizhou, People’s Republic of China; 4 Center for Injury Research and Policy & Center for Pediatric Trauma Research, The Research Institute at Nationwide Children’s Hospital, Columbus, OH, USA

**Keywords:** Burn, Children, Adult, Length of hospital stay, Total body surface area

## Abstract

**Objective:**

Burn wounds are a global public health problem, and a large number of casualties are caused by burns each year. In this study, we explored the epidemiological characteristics associated with burns and the factors affecting the length of hospital stay (LOS) in children and adults with burn wounds.

**Methods:**

Records of patients with burns in the Affiliated Hospital of Zunyi Medical University from January 1, 2014 to August 31, 2016 were retrieved. Information on demographic characteristics, mechanism of burns, first treatment received, clinical details of burns, and LOS were extracted from hospital medical records.

**Results:**

A total of 465 children and 327 adults with burns were identified over about 2.5 years. The ratio of male to female children with burn wounds was 1.6 and 2.3 in adults. The epidemiological characteristics of burns, including gender, season, location, etiology, degree and site, differed between children and adults. There were differences in external remedies used (e.g., traditional Chinese medicine powder) and wound protection (towels covering wounds) between children and adults, but few patients had both protected wounds and did not use external remedies. LOS was reduced with age (hazard ratio [HR] = 0.993) and skin grafting (HR = 0.339). LOS increased with the male gender (HR = 1.234), deep partial thickness burns (HR = 3.128), and full-thickness burns (HR = 1.791). LOS was increased when total body surface area (TBSA) of burns reached from 10% to 29% (HR = 3.978), when TBSA was greater than 30% (HR = 1.787), and heat vs. non-heat etiologies (HR = 1.497).

**Conclusion:**

Our findings for the epidemiological characteristics of burns in children and adults in Zunyi will help with a targeted awareness campaign and improve knowledge of first-aid and wound treatment.

## Introduction

Burn wounds are a global public health problem. A 2016 report by the World Health Organization indicated that an estimated 180,000 deaths annually were caused by burns (World Health Organization, 2018). Burns occur especially in developing countries, and almost two-thirds of burn incidents occur in African and Southeast Asian regions (World Health Organization, 2018).

A study from a burn center in Iran showed a mean length of hospital stay (LOS) of 9.04 days, and the average LOS of patients over 10-years-old was longer than those under 10-years-old (Karami et al., 2012). The elderly are at higher risk of injury than the younger age groups because they are more prone to injury (Peck, 2011). In China, research by Yao et al. (2011) on military hospitals showed that the average LOS for patients under the age of 16 was shorter than those over 16-year-olds. A study of the burden of burns in China showed that children and the elderly are impacted most by burns (Jin et al., 2017). A study on northern Guangdong Province showed that the type and cause of burns differs between children and adults (He et al., 2014).

Children and adults differ not only in their physical activities but also in their daily living and working environments. An evaluation of the epidemiological characteristics associated with burn patient groups can help in formulating prevention and treatment measures.

Zunyi city is located in southwest China at an altitude of 800–1,300 m. This results in a low atmospheric temperature in winter averaging 6.3 °C. Low income households often warm themselves using coal stoves. Therefore, we assumed that burn injuries in Zunyi may be unique compared to non-fatal injuries in other rural areas (Shi et al., 2014). Moreover, in Zunyi we also notice that there are quite a few different epidemiological relationships between adults and children, which seldom occur in other areas. For instance, the working environment is relatively poor for adults, with few or no occupational protection facilities (Li, 2013). Children are commonly poorly supervised by their grandparents, as most of their parents have to work far away from their hometown.

The Affiliated Hospital of Zunyi Medical University is the biggest hospital and coverage about 8 million residents and 500,000 migrant workers, and the unique burns treatment center in this region is located in this hospital. The hospital can treat most burns, including mild, severe, and severely burnt patients. Other hospitals have burn departments with a much smaller operating capacity.

In this study, we systematically analyzed the clinical data for patients in the largest hospital in Zunyi. We investigated differences in epidemiological characteristics between children and adults with burns, and then analyzed factors affecting LOS. We aimed to identify the differences in epidemiology among age groups to provide the government with factual data for prevention and treatment of burns. The government can formulate targeted policies through education and finance to protect the population at risk and reduce the burden of burns.

## Participants and Methods

### Participants

This study used the Chinese version of the *International Classification of Diseases* (ICD-10), codes T20 to T31, to retrieve medical record data from burn patients. T20–T30 represented the codes for different burn sites and T31 represented the code for different burn areas. We retrieved data from burn patients who were hospitalized for the first time at the Affiliated Hospital of Zunyi Medical University from January 2014 to August 2016.

### Data collection

The retrospective questionnaire collected data from medical records on general characteristics (age, gender, nationality, place of residence), clinical situation of burns (etiology, total body surface area (TBSA), site and degree of burns, any complications, any skin grafting, prognosis, limb activity after wound healing, LOS), the basic situation of the burn injury (where and what time the burn occurred, activity when the burn occurred, whether the burn was unintentional or intentional, etc.), and first aid after the burn (cold water rinse and rinsing time, external drugs used, protecting the wound with towels, etc.). Incomplete data were supplemented by contacting the patient by phone. This study was approved by the institutional review board of Zunyi Medical University (No. [2015]1-003).

### Discharge criteria and “survival time”

The standard for discharging burn patients was as follows: (1) vital signs were stable, (2) the wound had healed, or (3) the area of unhealed wound was <2 × 2 cm, clean with no infection, the skin was growing, and the patient or family members could continue the treatment after discharge from the hospital. “Survival time” was defined as the time from when the patient was admitted to hospital for treatment to the time the patient was discharged. Doctors determined that a patient could be taken care of at home with low risk of infection after observing and understanding the wound, based on past clinical experience. Censored data in this paper means that the patient’s condition had not reached the discharge standard, patients or their families required an early discharge, or that patients died during hospitalization.

The LOS was defined as the time period between the admission date and the discharge date recorded on the first page of the patient’s medical record.

The TBSA was the percentage of the burnt area of the skin in the whole-body surface area. The rule of Chinese nine method combined with the rule of palm method were used to estimate TBSA.

According to the month of hospital admission, we divided the year into four seasons: spring (March to May), summer (June to August), autumn (September to November), and winter (from December to February).

According to the types of occupation that the population in the area of residence was mainly engaged in, the place of residence category was divided into city (mainly engaged in commercial activities), town (mainly engaged in commercial and agricultural activities), and countryside (mainly engaged in agricultural activities).

The etiology of burns was categorized as hot liquid (including boiling hot water, hot oil, hot soup), fire-related factors (including fire, hot metals), electricity (including electric arcs, high voltage, and low voltage), and other etiologies (such as chemicals). In order to make our results more concise, we also defined a binary classification of etiology as heat etiology (including hot liquid, fire, hot solids, and hot gases), and non-heat etiology (including chemicals and electricity) in the Cox regression.

The degree of burns was categorized as superficial partial thickness (including dermal papilla and epidermis), deep partial thickness (involving the dermal reticular layer, but with an in-tact deep dermal structure), and full thickness (including subcutaneous fat, muscle, nerves and bones) (Huang, 2010).

Classification of burned site was divided into the following categories: head and neck, trunk (including buttocks and perineum), upper limbs, lower limbs, and organs (including respiratory tract, digestive tract, cornea.).

Inhalation injury refers to hot water, steam, flames, toxic fumes, or chemical poisons inhaled into the body, resulting in damage to the oral cavity, pharynx, larynx, airways, and even the lungs. Wound protection is defined as the actions that patients or their family’s take toward the burn wound, including the use of soft and clean towels, clothing gauze, and so on. This does not include the wound dressings or antimicrobial dressing systems applied by doctors.

### Statistical analysis

Epidata 3.0 (EpiData Association, http://www.epidata.dk/, Denmark) was used to build this study’s database. The Chi-square test was used to compare differences in categorical data, and the Kaplan–Meier method was used to examine factors affecting LOS, and factors possibly affecting LOS in Kaplan–Meier analysis were entered into the Cox proportional-hazards model. The Cox proportional-hazards regression model can take full advantage of incomplete censored datasets, also including LOS in the model. Hazard ratios (HR) were estimated by taking the ratio of the risk function of the exposure group to the non-exposed group at a given time, and calculating 95% confidence intervals (CIs). All statistical analyses involved using SPSS 18.0 (SPSS Corp., Chicago, IL, USA). All tests were two-sided, with *P* < 0.05 considered statistically significant.

## Results

### Characteristics of children and adults with burns

We collected data from three hospital departments (plastic surgery, ophthalmology, gastroenterology) for 792 inpatients with burns: 465 children with an average age of 3.61 ± 3.57 years, and 327 adults averaging 42.48 ± 14.76 years. There were 14 severe cases where patients died however the other 778 patients were survivors. Both children and adults were dominated by males. There were 1.6 times more male children and 2.3 times more male adults with burns compared to females, and the incidence of burns in adult males was higher (*P* = 0.020) (Table 1). The overall median LOS was 9 days (range 5–19), children 8 days (range 5–15), and adults 12 days (range 6–27). A total of 195 child burns (42%) occurred in winter, 121 child burns (26%) occurred in spring. A total of 111 adult burns (34%) occurred in summer, and 86 adult burns (26.3%) occurred in winter (*P* < 0.001) (Table 1). The home was the main place of burns for children (86.0%), whereas adult burns occurred more frequently in the workplace (44.6%), followed by the home (*P* < 0.001). A total of 328 children burn cases (70.5%) occurred while playing and 256 adult burn cases (78.3%) occurred during work (*P* < 0.001). Nationality and residence were not statistically significant for either children or adults (Table 1).

**Table 1:** Characteristics of children and adults hospitalized with burns.

Variable	Children (*n*_1_ = 465)	Adult (*n*_2_ = 327)	Chi-square	*P*-value
Gender			5.409	0.020
Male	287 (61.7)	228 (69.7)		
Female	178 (38.3)	99 (30.3)		
Ethnicity			0.033	0.857
Han	438 (94.2)	309 (94.5)		
Minority	27 (5.8)	18 (5.5)		
Residence			0.724	0.696
City	68 (14.6)	55 (16.8)		
Town	67 (14.4)	47 (14.4)		
Countryside	330 (71.0)	225 (68.8)		
Season			34.896	<0.001
Spring	121 (26.0)	83 (25.4)		
Summer	81 (17.4)	111 (34.0)		
Autumn	68 (14.6)	47 (14.4)		
Winter	195 (42.0)	86 (26.3)		
Location of burn			242.238	<0.001
Home	400 (86.0)	142 (43.4)		
School or workplace	5 (1.1)	146 (44.6)		
Others	60 (12.9)	39 (12.0)		
Activity during burn			501.818	<0.001
Eating	50 (10.8)	7 (2.1)		
Playing	328 (70.5)	33 (10.1)		
Work	13 (2.8)	256 (78.3)		
Other	74 (15.9)	31 (9.5)		

**Notes:**

Data are *n* (%).

Season: spring (March to May), summer (June to August), autumn (September to November), and winter (from December to February).

### Clinical manifestations of burns in children and adults

The etiology of burns in children was mainly hot liquid, followed by fire-related factors, and the etiology for adults was diverse, the main cause being fire-related factors, followed by electricity (*P* < 0.001). Among both children and adults, most burns were multi-site burns. Children most frequently had trunk burns, followed by lower-limb burns. Adults frequently had upper limb burns, followed by lower limb burns (*P* < 0.001).

Deep partial thickness burns were predominant in both children and adults, with superficial partial thickness burns being the second most common for children, and full-thickness burns second most common for adults (*P* < 0.001). More adults than children had inhalation injury and skin grafting (*P* < 0.001) (Table 2). Protecting the wound with towels was more frequent with children than adults (21.1% vs. 11.3%; *P* < 0.001). Children and adults did not differ in cold-water rinsing after burns (*P* = 0.847), external remedy used (*P* = 0.122), or TBSA (Chi-square = 5.939, *P* = 0.051) (Table 2).

**Table 2:** Clinical manifestations and first remedy of children and adults with burns.

Variable	Children (*n*_1_ = 465)	Adult (*n*_2_ = 327)	Chi-square	*P*-value
Etiology			262.306	<0.001
Hot liquid	357 (76.8)	65 (19.9)		
Fire-related	68 (14.6)	129 (39.4)		
Electricity	15 (3.2)	88 (26.9)		
Others	25 (5.4)	45 (13.8)		
TBSA			5.939	0.051
<10%	308 (66.2)	229 (70.0)		
10–29%	138 (29.7)	76 (23.2)		
≥30%	19 (4.1)	22 (6.8)		
Burn site			43.766	<0.001
Head and neck	236 (50.8)	138 (42.2)		
Trunk	322 (69.2)	146 (44.6)		
Upper limbs	230 (29.5)	197 (60.2)		
Lower limbs	263 (56.6)	175 (53.5)		
Organ^⋆^	23 (4.9)	46 (14.1)		
Degree of burn			57.965	<0.001
Superficial partial thickness	138 (29.7)	78 (23.9)		
Deep partial thickness	285 (61.3)	151 (46.2)		
Full thickness	42 (9.0)	98 (29.9)		
Skin grafting			12.891	<0.001
Yes	34 (7.3)	50 (15.3)		
No	431 (92.7)	277 (84.7)		
Inhalation burn			21.444	<0.001
Yes	12 (2.6)	34 (10.4)		
No	453 (97.4)	293 (89.6)		
External remedy used			9.527	0.023
Drugs*	35 (7.5)	34 (10.4)		
Toothpaste, egg-white, etc.	17 (3.7)	2 (0.6)		
Others	10 (2.2)	9 (2.8)		
No remedy	403 (86.6)	282 (86.2)		
Wound protection			12.935	<0.001
Simple bandage	98 (21.1)	37 (11.3)		
Not bandaged	367 (78.9)	290 (88.7)		
Cold water rinsing			0.037	0.847
Yes	20 (4.3)	15 (4.6)		
No	445 (95.7)	312 (95.4)		

**Notes:**

Differences in etiology, burn site, degree of burn, skin grafting, inhalation burn, external remedy used and wound protection between burnt children and adults were statistically significant.

Data are *n* (%).

TBSA, total body surface area.

⋆ Including eyes and annexa oculi, respiratory tract, other internal organs.

* Including anti-infective drugs and traditional Chinese medicine.

### Factors affecting LOS by Kaplan–Meier method

The univariate analysis Kaplan–Meier method showed that inhalation burns affected LOS for children but not adults. TBSA, degree of burn, and complications (including shock, organ damage, and wound infection) affected LOS for both children and adults (Table 3).

**Table 3:** Kaplan–Meier analysis of factors associated with length of hospital stay (LOS) in children and adults with burns.

Variable	Children			Adults		
	Median LOS, days	Chi-square*	*P*-value	Median LOS, days	Chi-square*	*P*-value
Gender		0.69	0.406		0.07	0.788
Male	10			19		
Female	11			17		
TBSA		67.04	<0.001		28.47	<0.001
<10%	7			14		
10–29%	16			25		
≥30%	41			42		
Degree of burn		80.62	<0.001		63.83	<0.001
Superficial partial thickness	6			9		
Deep partial thickness	11			17		
Full thickness	33			36		
Etiology		6.98	0.072		5.66	0.129
Hot liquid	10			15		
Fire-related	7			19		
Electricity	39			26		
Others	15			20		
Inhalation burn		6.10	0.013		0.88	0.349
Yes	22			25		
No	10			18		
Complications		23.30	<0.001		6.78	0.009
Yes	34			32		
No	9			17		
Medical treatment ≤24 h		0.53	0.467		0.02	0.893
Yes	10			19		
No	10			18		

**Notes:**

TBSA, degree of burn and complication were common factors that affected LOS in burnt children and adults, whereas inhalation burn affected only in burnt children.

* Log-rank test.

### Factors affecting LOS by Cox proportional-hazards regression analysis

Factors reducing LOS were age (HR = 0.993, CIs: 0.988–0.997) and the use of skin grafting (HR = 0.339, CIs: 0.254–0.451). Conversely, factors increasing LOS were male gender (HR = 1.234, CIs: 1.026–1.484), deep partial thickness burns (HR = 3.128, CIs: 2.349–4.166), full-thickness burns (HR = 1.791, CIs: 1.379–2.327), TBSA ranging from 10% to 29% (HR = 3.978, CIs: 2.551–6.205), TBSA ≥ 30% (HR = 1.787, CIs: 1.135–2.813) and heat vs. non-heat etiology (HR = 1.497) (Table 4).

**Table 4:** Cox proportional-hazards regression analysis of factors affecting LOS in patients with burns.

Variable	Variable assignment	B	SE	Wald	*P*-value	HR (95%CI)
Age	Continuous variables	−0.008	0.002	10.461	0.001	0.993 (0.988–0.997)
Gender	1 = Male; 0 = Female	0.210	0.094	4.976	0.026	1.234 (1.026–1.484)
Degree of burn	Dummy variable			66.404	<0.001	
Superficial partial thickness						1.00
Deep partial thickness		1.140	0.146	60.851	<0.001	3.128 (2.349–4.166)
Full thickness		0.583	0.134	19.037	<0.001	1.791 (1.379–2.327)
TBSA	Dummy variable			81.342	<0.001	
<10%						1.00
10–29%		1.381	0.227	37.078	<0.001	3.978 (2.551–6.205)
≥30%		0.581	0.231	6.292	0.012	1.787 (1.135–2.813)
Etiology	1 = Heat; 0 = non-heat*	0.404	0.118	11.770	0.001	1.497 (1.189–1.886)
Skin grafting	1 = Yes; 0 = No	−1.083	0.147	54.553	<0.001	0.339 (0.254–0.451)
Inhalation burn	1 = Yes; 0 = No	0.073	0.204	0.127	0.721	1.076 (0.721–1.605)
Complications	1 = Yes; 0 = No	−0.199	0.162	1.518	0.218	0.819 (0.597–1.125)
Medical treatment ≤24 h	1 = Yes; 0 = No	−0.032	0.112	0.083	0.773	0.968 (0.777–1.207)

**Notes:**

Gender, TBSA, degree of burn and etiology were risk factors for LOS, while age and skin grafting were protective factors of LOS.

B, regression coefficients; SE, standard error; HR, hazard ratio; CI, confidence interval.

* Heat etiology including hot liquid, fire, hot solids, and hot gases; all other factors were non-heat etiology.

## Discussion

Prior to this study, it was not clear if different treatment and prevention strategies are needed for children and adult burn patients. Our study found the epidemiological characteristics of burns including gender, season, location, etiology, degree and site, differed between children and adults. LOS was reduced with age and skin grafting. LOS was increased if the patient was male, if deep partial thickness and full-thickness burns were present, if the TBSA was from 10% to ≥30%, and if the burns were caused by heat. Our findings can help with a targeted prevention campaign and to improve knowledge of first-aid, policy and care of burns for the public. Specific interventions for children and adults might potentially include actions to provide assistive devices and modify hazardous environments.

The ratio of male to female children with burns was 1.6 in children and 2.3 in adults. According to the tenth annual demographic for Zunyi City in 2016, the ratio of male to female was 1.1, so the gender ratio of children and adults with burns is higher than that of the general population in Zunyi. This suggests that burns are frequent in males, which agrees with some reports (Nthumba, 2016; Jiang et al., 2010; Latifi & Karimi, 2017). Previously, however, the incidence of burns was found higher for males than females only in adults, with no gender difference in children (Blom et al., 2016). Furthermore, another study reported burn incidence higher in females than males (Parray et al., 2015). The gender ratio leaning toward males was greater for adults than children with burns, so adult men are more likely to experience burns. Adult males, who often work outdoors, are likely to be involved in a poor working environment, with little or even no occupational protection facilities. Moreover, in China, males are the main economic resource of the family, and their employment is much higher than that of women, so their total occupational exposure is greater than for females. Adult males should be the target of public safety education to reduce the incidence of burns.

In Zunyi, the top incidence of burns for children was in the winter, whereas for adults, it was in the summer. For children, this finding may relate to the low temperature in winter without central heating systems. When residents depend on coal stoves for heating and cooking, it is easy for children’s clothes to be ignited when they wander near the stove. Moreover, in many local residences, boiled hot water is first added to the wash basin before adding cold water, creating a burn hazard for active children. Finally, the Spring Festival holiday is the largest traditional Chinese festival in the winter, when children are frequently hospitalized for injury due to firecrackers and fireworks (Lin et al., 2012). Young adults are exposed to a large number of occupational risk factors (Ortiz-Prado, Luciana & Iturralde, 2015). In summer, minimal clothing cannot effectively protect the body against injury. In our survey, hospitalized children were mostly preschool children, with the home being their primary location, and playing their primary activity. This study suggests children are burned mostly when they play at home. Adults are exposed to risk factors of burns in the workplace, but the home is also an important source, with cooking and heating flames and hot food (or water), as well as burning charcoal, being common family risk factors.

Hot liquid was the main etiology of burns in children, which is similar to the Liu et al. study (Liu et al., 2012). The common risk factors of burns in the home are the kitchen, which includes hot food, hot water, fire, and especially hot liquids. With the improvement in the economy, some families use drinking water dispensers, and children use the water valve as a toy, releasing hot water which easily burns the hand (Zhu, 2012). The depth and condition of burns will be more serious in children because the skin of children is thinner than adults. The main etiology for adult burns is fire-related; adults are in contact with fire mainly from coal, gas, and charcoal in their workplace. Although the TBSA caused by high voltage electricity burns is small, the degree of electrical burns is mostly full-thickness. Even nerves, blood vessels and muscles are damaged by electrical burns, and many need escharectomy and skin grafting or a pedicle flap transplant. The prognosis is often poor, with high chance of resulting in a disability.

The skin graft rate was lower for children than for adults with burn wounds. The main assumption being that children’s skin grows faster than adult’s (Huang, 2010). Burn sites in children and adults were in multiple areas; the most common burn site in children was the trunk and the most common in adults was the upper limb, perhaps because of their work. Our results are similar to some previous studies in that no specific site was clearly the most commonly burned (Akita et al., 2005; Taira et al., 2010). Liu’s (2012) study showed that inhalation injury had a cumulative effect on the burn condition. The main etiology for adults was a fire-related factor, which is more likely to cause inhalation injury than other etiologies.

The most effective treatment after a burn is cold-water flushing more than 20 min but without external medicines (Wood et al., 2016). The pastes for external application can not only deepen the wound but also hamper its evaluation and treatment (Yoon et al., 2016). In our study, there were some differences between children and adults in the external remedy used for wound protection. Self-treatments after burns were not satisfactory. Health education for burns is weak in the Zunyi area, and the government should publicize correct knowledge about burns in schools and communities. Children’s wound protection rate is higher than adults, possibly because most children experience burns in winter and the wound is covered to protect against the cold weather.

The overall median LOS was 9 days (range 5–19), similar to the Cornet et al. findings (Cornet et al., 2017). The median LOS for children was 8 days (range 5–15) and for adults 12 days (range 6–27). LOS is also the result of the interaction of various influencing factors. Multivariate analysis showed that skin grafting (HR = 0.339), and age (HR = 0.993) were protective factors for LOS. A possible reason why age reduced LOS is that organ function gradually improves with age, so repair will be faster in adults. Moreover, the nutrients absorbed by burned children are divided between growth needs and healing the wound; adults grow less and can focus their energy on healing the wound. However, the HR was very close to 1, which suggests age was not the main influencing factor of LOS. Sometimes, the skin of the elderly is shrinking and thinning, and deep burns are even prone to occur (Zhou, Tang & Xue, 2016). Weakening of physical functions of the elderly can lead to slow healing of wounds after burns (Jiang, Min & Guo, 2017). Skin grafting can reduce LOS because skin grafting can cover the wound quickly and grow faster than the natural wound condition. When the transplanted skin survives and the vital signs are stable, the patient can be discharged from the hospital. The male gender was a risk factor for burns; boys like to explore strange environments and new things, and male adults are more easily exposed to risk factors at work.

The risk of LOS was higher for deep partial thickness burns than for full-thickness burns, HR = 3.128 and HR = 1.791, respectively. This may be due to the patient’s neuromuscular involvement in the full-thickness skin. Most patients are treated by pedicle flap transfer. When the patient’s vital signs are stable and the flap model survives, the patient is discharged. TBSA from 10% to 29% had the greatest impact on LOS (HR = 3.978). An increase in TBSA causes a more serious the wound exudation. This disturbs the internal environment of the wound and other systems are damaged. The condition is more complicated and patients need to be treated for a long time. Moreover, the larger the area of skin damage, the longer it takes to grow skin. TBSA ≥30% was also a risk factor (HR = 1.787), but was less than TBSA from 10% to 29%. Some burn patients may have died within a few days of hospitalization due to multiple organ dysfunction syndromes. Heat etiology was a risk factor (HR = 1.497) possibly because heat damage is not only due to direct contact with the skin but also damages deep tissue.

## Conclusion

We compared the situation between children and adults hospitalized for burn wounds and explored the different mechanisms and factors that may help prevent burns. Burn prevention for the entire population not only needs support from government, but also needs families, schools and factories to participate in the burn prevention process. Therefore, there is a need to establish a family-school-factory-government prevention system to reduce the occurrence of burns.

## Supplemental Information

10.7717/peerj.5740/supp-1 Supplemental Information 1 Raw data for burnt children and adults. Original data including demographics, first aid and clinical data.

## Additional Information and Declarations

### Data Availability

The following information was supplied regarding data availability: The raw data are provided in a Supplemental File.
